# Multifaceted Roles of AFG3L2, a Mitochondrial ATPase in Relation to Neurological Disorders

**DOI:** 10.1007/s12035-023-03768-z

**Published:** 2023-11-28

**Authors:** Ranita Ghosh Dastidar, Saradindu Banerjee, Piyush Behari Lal, Somasish Ghosh Dastidar

**Affiliations:** 1 Department of Biochemistry, Kasturba Medical College, Manipal, Manipal Academy of Higher Education, Manipal, Madhava Nagar, Manipal, 576104 Karnataka India; 2 Centre for Molecular Neurosciences, Kasturba Medical College, Manipal, Manipal Academy of Higher Education, Manipal, Madhava Nagar, Manipal, 576104 Karnataka India; 3 Department of Microbiology, Kasturba Medical College, Manipal, Manipal Academy of Higher Education, Manipal, Madhava Nagar, Manipal, 576104 Karnataka India

**Keywords:** AFG3L2, Zinc metalloprotease, SCA28, Ataxia, Neurological disorders, Mitochondria

## Abstract

AFG3L2 is a zinc metalloprotease and an ATPase localized in an inner mitochondrial membrane involved in mitochondrial quality control of several nuclear- and mitochondrial-encoded proteins. Mutations in *AFG3L2* lead to diseases like slow progressive ataxia, which is a neurological disorder. This review delineates the cellular functions of AFG3L2 and its dysfunction that leads to major clinical outcomes, which include spinocerebellar ataxia type 28, spastic ataxia type 5, and optic atrophy type 12. It summarizes all relevant *AFG3L2* mutations associated with the clinical outcomes to understand the detailed mechanisms attributable to its structure-related multifaceted roles in proteostasis and quality control. We face early diagnostic challenges of ataxia and optic neuropathy due to asymptomatic parents and variable clinical manifestations due to heterozygosity/homozygosity of *AFG3L2* mutations. This review intends to promote AFG3L2 as a putative prognostic or diagnostic marker.

**Graphical Abstract:**

Functions, mutations, and clinical manifestations in AFG3L2, a mitochondrial AAA + ATPases.

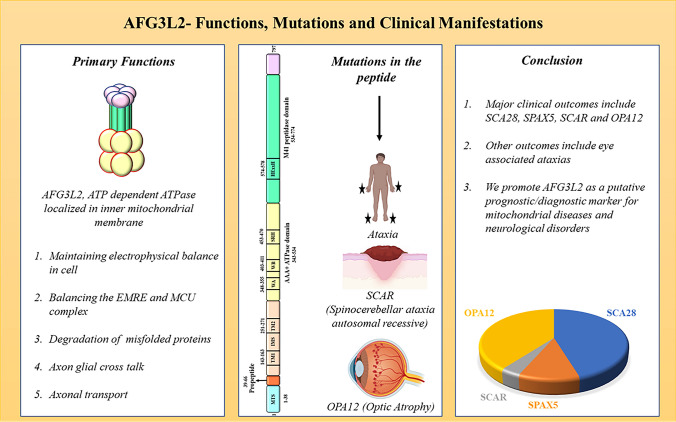

## Introduction

Mitochondria, as an organelle, plays a vital role in numerous life-sustaining activities in eukaryotic cells, for example, adenosine triphosphate (ATP) synthesis, calcium homeostasis, and beta-oxidation of fatty acids [1, 2]. Regulated proteolysis of mitochondrial proteome is essential for proper mitochondrial function. Alteration in the mitochondrial protein level may adversely impact energy production, calcium signaling, and other activities in eukaryotic cells leading to various diseases such as cardiovascular diseases and late-onset neurodegenerative diseases [3–6]. Deregulation of mitochondrial proteome is correlated with mitochondrial diseases such as dominant optic neuropathy, Parkinson’s disease (PD), Alzheimer’s disease (AD), Huntington’s disease, sarcopenia, and bipolar disorder [7–9]. Several proteases play an important role in maintaining mitochondrial proteostasis. One example of a protease that is involved in mitochondrial proteostasis is AFG3L2. AFG3L2 is a mitochondrial ATPase associated with diverse cellular activities (AAA) protease, which is found in the inner membrane of mitochondria [10]. It can function solely as a homo-hexamer or in combination with a protease, paraplegin (encoded by SPG7), in the form of a hetero-hexamer [11]. AFG3L2 has a varied role in cellular physiology. It is involved in the axonal anterograde transport of mitochondria [8], mitochondrial protein synthesis, and cellular respiration [12]. AFG3L2 mutations cause three monogenic disorders which are spinocerebellar ataxia type 28 (SCA28), spastic ataxia type 5 (SPAX5), and optic atrophy type 12 (OPA12) and have been reported for their mechanistic association with other diseases such as PD and other eye associated ataxias like ophthalmoparesis and oculomotor apraxia [9]. It is involved in steroid synthesis regulation via StAR overload response (SOR) [13–23]. This review discusses the multifaceted roles of AFG3L2 in mitochondrial physiology and its role primarily in spinocerebellar ataxia type 28 (SCA28) and other diseases.

## AFG3L2: an Inner Mitochondrial Membrane ATPase

AFG3L2 is an inner mitochondrial membrane (IMM) ATPase and a zinc metalloprotease. YME1L, OMA1, and paraplegin are a few other examples of mitochondrial ATPases. These proteases are located in the inner mitochondrial membrane. In brief, OMA1, YME1L, AFG3L2, and paraplegin are the mitochondrial AAAs that play a vital role in mitochondrial proteostasis in their active oligomeric forms. AAA proteases located in the IMM are categorized into ATP-dependent and ATP-independent proteases. These proteases, including AFG3L2, are ubiquitously expressed [11]. They are the descendants of highly conserved bacterial FtsH AAA zinc metalloproteases and are sub-grouped as ring-shaped P-loop NTPases [11]. They are also called as the M41 family of zinc metalloproteases [20].

### Location and Structure

AFG3L2 is located in the matrix side of IMM (m-AAA) [11]. Other mitochondrial AAAs that play a vital role in mitochondrial proteostasis in their active oligomeric forms are YME1L and OMA1 [11]. They are located in the IMM side of mitochondria (i-AAA). YME1L (Yme1p in yeast) is an ATP-dependent protease while the OMA1 is an ATP-independent protease and both function in their homo-hexameric form [21].

AFG3L2 is an m-AAA that co-localizes and interacts with another m-AAA, paraplegin. To maintain mitochondrial proteostasis, AFG3L2 can function in both homo-hexamer and hetero-hexameric forms with itself or paraplegin [11]. The human gene encoding *AFG3L2* was mapped at chromosome 18p11 by Brunella Franco’s laboratory. A mutant variant of *AFG3L2* that causes SCA28 was mapped in the SCA locus on chromosome #18 at 18p11.22-q11.2 by Alfredo Brusco’s laboratory in 2006 [24–26]. Nuclear encoded *Afg3l2*, on translocation to the mitochondria, undergoes processing by MPP peptidase followed by further autocatalysis for its maturation [27]. Unlike paraplegin, no isoform of AFG3L2 has been identified till date that is localized outside mitochondria, like in ER, suggesting that hetero-oligomers can be formed only in mitochondria [28]. Hexamers are solely stabilized by ATP binding [29]. Although there is variability in the assembly of murine m-AAA proteases that is largely dependent on the availability of the individual subunits, the hetero-hexamer form of Afg3l2 and paraplegin is prevalent in neuronal cells [30]. Kress and Weber-Ban have summarized the works of Augustin and co-workers that clearly depict the formation of these hexamers, their molecular determinants, and their roles in substrate dislocation from membranes. The ATP-bound hexamer subunits are considered active states. In the hexamer, three alternating ATP-bound subunits together connect with the substrate. The other three subunits remain in the inactive form, but because of ATP hydrolysis, they become active and take the charge of substrate. It has been reported that each subunit controls the ATPase activity of their neighboring subunits. The alternating hexamer states help in loop movements so that always either two or three subunits have the grip on substrate. Substrates can be detached from one subunit by the allosteric inhibitory effect of others. Subunits of hetero-hexamers of AFG3L2/paraplegin around the ATPase ring manipulate every alternating subunit and generate a firing pattern with two alternate groups. Respiratory impairment offers an in vivo read-out and is an indicator of deregulation of m-AAA protease activity [31, 32] that can be used as a diagnostic approach in mitochondrial diseases.

**Fig. 1:**
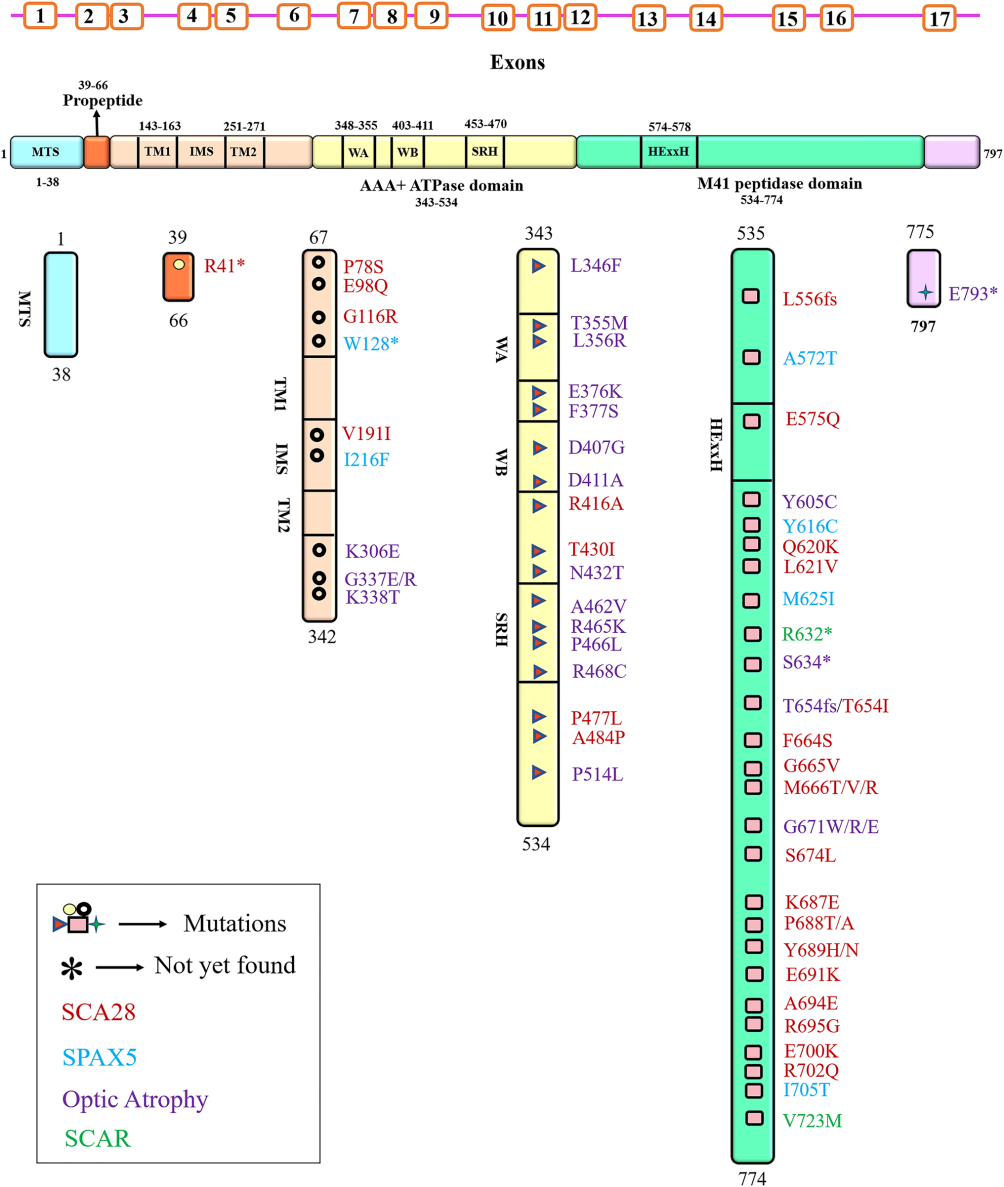
The genomic orientation and the domain structures of human AFG3L2 protein. The AFG3L2 protein consists of 17 exons and 797 amino acids. The gene position is 18p11.21 and a total of 48 kb. The figure represents various mutations that cause SCA28 (red), SPAX5 (blue), optic atrophy (purple), and SCAR (green)

**Fig. 2:**
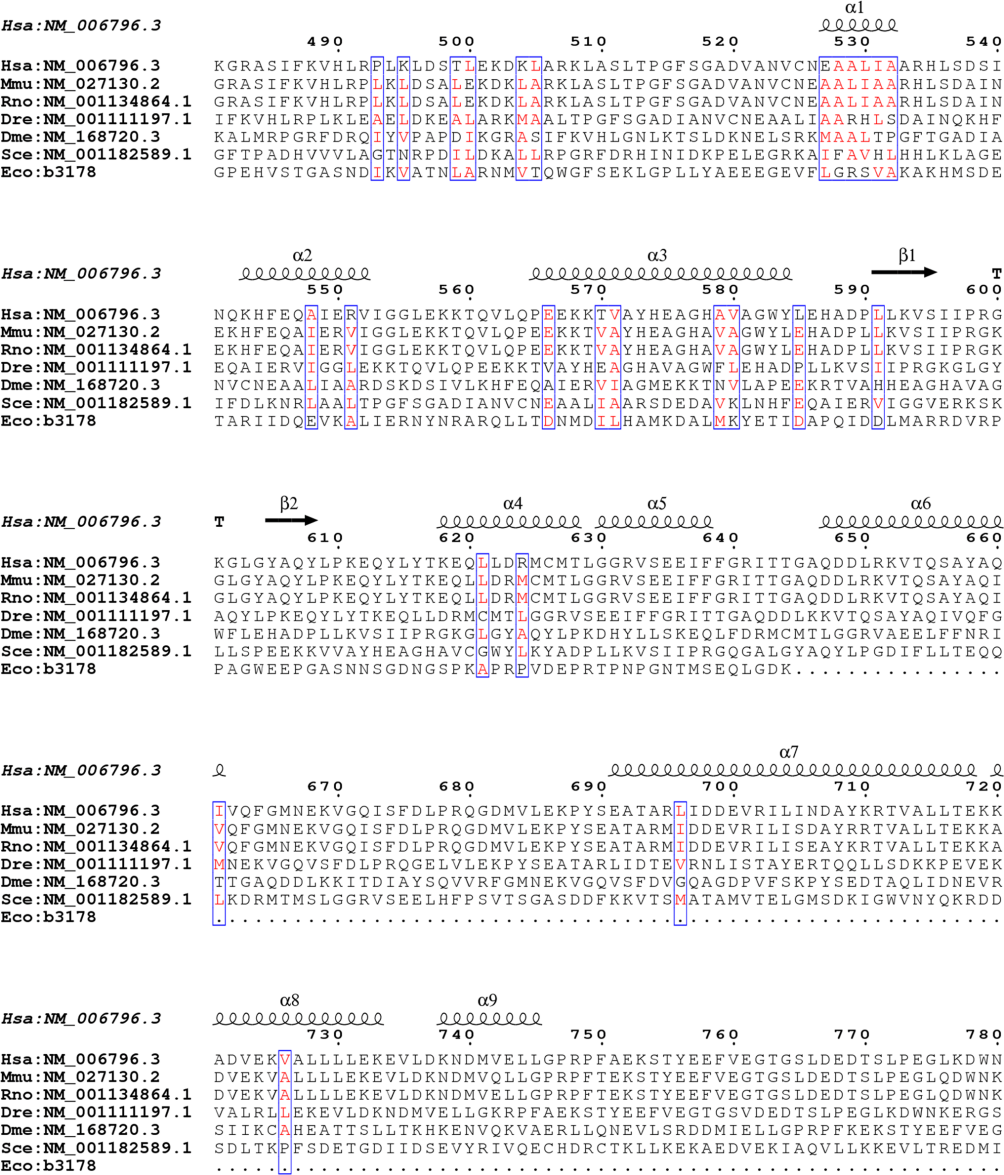
Alignment and substitutions of amino acids in the highly conserved regions of AFG3L2 protein in different study models from bacteria to humans. The alignment is depicted for regions of secondary structures of *Homo sapiens* (Hsa, human), *Mus musculus* (Mmu, mouse), *Danio rerio* (Dre, zebrafish), *Drosophila melanogaster* (Drm, fruit fly), *Saccharomyces cerevisiae* (Sce, yeast), and *E. coli* (Eco, bacteria). This alignment was done in ESPript 3.0 with PDB secondary structure information for similarity index with percent equivalency at a global score of 0.7. Blue boxes are highly conserved regions with all red-colored amino acids showing conservation across those organisms

AAA + domains are highly conserved with either classic cross-hatching clade or HCLR stippling clade [33]. m-AAAs include a least conserved N-terminal distal domain, two transmembrane spans, an AAA + ATPase module with walker A/walker B motif, the second region of homology (SRH) transmembrane spanning domain, and a HExxX or HxxEH or HExGH motif containing a C-terminal metalloprotease or proteolytic domain (Fig. 1) [11, 34]. Walker A motif is an ATP binding GX4GKT/S sequence, and walker B motif is hydrophobic (h), i.e., hDD/E sequence with transmembrane domains [33]. We have studied the conserved regions of AFG3L2 by alignment of amino acids of AFG3L2 protein from different organisms, bacteria to humans (Fig. 2). The highly conserved regions of AFG3L2 among different species are shown in blue (Fig. 2). On top of the amino acid sequences, alpha-helical regions are shown with coil structures, and beta-sheet regions are shown with right-sided arrows (Fig. 2). Glynn’s laboratory generated a reconstituted active AFG3L2 with intact protease activity by displacing the transmembrane domains with a CCHEX sequence stimulating hexamerization of the catalytic domains of the protease [29, 34]. Their work clearly depicts how AFG3L2 can maintain its high specificity and yet differentiate between substrates by identifying degron sequences accessible for peptide bond cleavage. Recently, Puchades and co-workers have elaborated a detailed cryo-EM structure of a truncated construct of AFG3L2 comprising the ATPase and peptidase domains (core of AFG3L2, residues 272–797). For a good understanding of the unique characteristics of the stability and activity of AFG3L2, they also showed detailed structure of disease-specific mutations elucidating the structure–function relation of this m-AAA (discussed later in the *AFG3L2* mutation section). NMR structural analysis has revealed an intermembrane space domain of AFG3L2 that is located in the membrane periphery and its furthest region interacts with substrates and prohibitins as and when required [35] aiding in membrane stabilization. The C-terminal protease domain has evolved from the i-AAA YME1L that can only recruit soluble substrates to the m-AAA AFG3L2 where it recruits membrane-bound substrates by charged interactions hence maintaining distinct features for these ATPases. Sequential substrate processing steps include (i) substrate recruitment by N- and C-termini of ATPase domain, (ii) ATP-dependent substrate intercalation by pore loop 1 for translocation, (iii) unfolded substrate transfer by pore loop 2 and central protrusion chamber, and (iv) substrate cleavage at zinc-associated protease active site [36]. Like in other AAA + ATPases, pore-loop 1 forms an aromatic spiral staircase that interacts with the substrate and drives translocation. To adopt a membrane-proximal position, the C-terminus encircles the hexamer conferring a complex stability [27]. The substrate translocation is spatially facilitated by the tightly packed aromatic residues immediately in front of pore-loop 1, which provides a lucid configuration of a central channel. The substrate is further transferred to a central protrusion by pore-loop 2 within the protease domain. A staircase, like spirals in N-termini of ATPase domain, and pore loops 1 and 2 help in the hand-held translocation of substrate from this domain to the catalytic core [27]. These domains work in cooperation to drive nucleotide-driven allosteric changes and pull substrate through the central channel as ATP is hydrolyzed [36].

### Primary Functions

The primary functions of mitochondria include ATP production, fatty acid oxidation, and functioning as calcium ion reservoir [1, 2]. Nuclear-encoded mitochondrial proteins are synthesized in the cytosol and transported into the mitochondria, whereas the mitochondria-encoded proteins are facilitated for co-translational insertion in the inner membrane of mitochondria with simultaneous processing to enable efficient assembly into respective respiratory complexes within the mitochondria [37]. Mitochondrial-encoded proteins being hydrophobic require co-translational insertion into the inner membrane and further require quality control simultaneously for misfolded or truncated proteins [38]. The primary function of the mitochondrial proteases is to engage and process unprocessed, misfolded, and damaged polypeptides to maintain mitochondrial homeostasis. Mitochondrial proteostasis is brought out by protein degradation, partial processing with chaperonic rearrangements, or even relocalization of proteins to their accurate location within the mitochondria [34]. Mitochondrial proteases have pleiotropic functions that include protein import, quality control, protein processing, phospholipid trafficking, ribosome assembly, OXPHOS assembly, MCU complex assembly, mtDNA stability, mitophagy, apoptosis, and hypoxic response [4]. It is quite intriguing to understand the roles that mitochondrial proteases may play to influence varied cellular responses and might aid or abet a plethora of diseases that may range from cancer and neurological disorders to aging [39–43]. Proteostasis imbalance can be brought by a reduction in the capacity of protein folding and excessive protein aggregates. Mitochondrial unfolded protein response (mtUPR) generates a protective response against this imbalance [44]. Hetero-dimer of CHOP and C/EBPβ, family of CCAAT/enhancer binding proteins, activates the transcription of mtUPR responsive genes that includes genes encoding mitochondrial proteases YME1L1 and MPPβ [45]. These genes contain a CHOP element in their promoters. Due to the lack of CHOP element in both *Afgl32* and *Spg7*, these genes are not involved in mtUPR [45] although they play key role in mitochondrial protein folding.

### Mitochondrial Co-translational Protein Quality Control

AAA protease complexes are responsible for maintaining the mitochondrial quality control that includes homo- or hetero-oligomeric m-AAA proteases [30]. All 13 mitochondrial encoded OXPHOS proteins are co-translationally processed by AFG3L2 after the insertion of these nascent chains into the IMM by OXA1L/OXA1 (coding insertase enzyme) [38]. Genetic interaction of *AFG3L2*, *OXA1*, and F1F0 ATP synthase (complex V of OXPHOS), well documented in budding yeast Afg3l2p, regulates co-translational proteostasis that is quintessential in the maintenance of organelle homeostasis and affects the bioenergetics of a cell. Kah Ying Ng’s work has used MT-ATP6 as a representative substrate to understand this co-translational quality control and delineate the steps of regulation in MT-ATP6 pathogenic variants [38]. *AFG3L2* is involved in the quality control of OXA1L-mediated *MT-ATP6* insertion in IMM. Loss of function of AFG3L2 affects the mitochondrial morphology and simultaneously critical pathways leading to mitochondrial gene expression impairment.

### Protein Aggregates

Many neurodegenerative diseases result from protein misfolding leading to aggregation. m-AAAs help in keeping a balance by maintaining the proteostasis. For example, in a genome-wide RNAi screen, *AFG3L2* came up as a gene involved in the suppression of mutanthuntingtin protein accumulation in the mitochondria, thus indicating therapeutic potential [17].

### Steroid Synthesis

StAR overload response (SOR) is the process of enrichment of mitochondrial proteases and their transcripts by steroidogenic acute regulatory protein (StAR) expression. StAR regulates steroid synthesis in adrenal cortex and gonads by activating the CYP11a1/P450scc enzyme in the OMM. StAR expression also increases the gene transcription of *LON* and *AFG3L2/SPG7* [46, 47]. Excessive StAR production leads to a sequential degradation process of StAR, due to overload, in the OMM by LON followed by in the IMM where it is degraded by LON and AFG3L2/paraplegin. This avoids SOR in the mitochondrial matrix [47].

### Functions Related to Expression

**Fig. 3:**
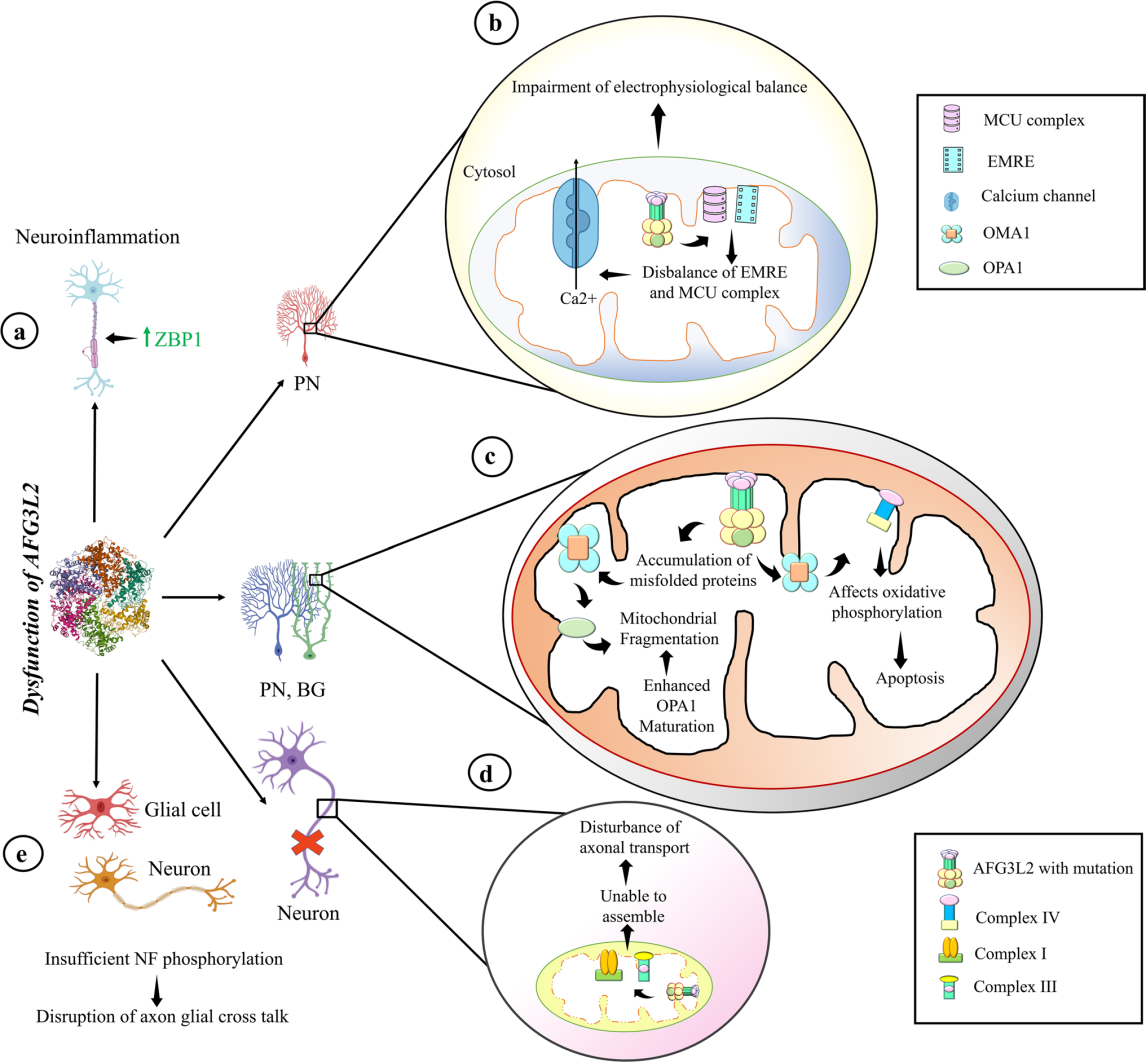
Biological pathways that are affected due to mutations and dysfunction of AFG3L2 causing SCA28. (**a**) The mutation of *AFG3L2* is responsible for increasing the neurotropic factor ZBP1 that in turn causes neuroinflammation. (**b**) MCU and EMRE, two complexes found in the mitochondrial inner membrane, can be disrupted by dysfunctional AFG3L2, which leads to disruption of electrophysiological balance by releasing excess Ca^2+^ from the mitochondrial matrix to the cytosol. (**c**) Mutations in *AFG3L2* generate an accumulation of misfolded proteins in the mitochondrial matrix followed by the activation of OMA1 protease which enhances the OPA1 processing to its short form and causes the mitochondrial fragmentation. On the other hand, the Complex IV of electron transport chain (ETC) is hampered because of *AFG3L2* mutation. (**d**) The dysfunctional AFG3L2 inhibits the assembly of Complex I and Complex III of ETC that finally leads to the disruption of axonal transport. (**e**) The defective AFG3L2 is responsible for the disbalance of axon glial cross talk due to insufficient neurofilament. PC, Purkinje cells; BG, Bergmann glial

*AFG3L2* is ubiquitously expressed. It is evident from previous studies that *Afg3l2* is expressed throughout the mouse brain corroborating its essential role in the function of the neurons [19]. Further enhanced expression is observed in the large cell body containing neurons that include brainstem motor neurons, mitral cells of olfactory bulb, pyramidal cells of hippocampus and neocortex, Purkinje, and deep nuclei cells of cerebellum [19, 48]. However, *Afg3l2* expression patterns are not strictly related to the occurrence of SCA28 or SPAX5 development [19]. So, it can be predicted that there might be unidentified proteins regulated by AFG3L2 which can be a participant to the SCA28 or SPAX5 pathophysiology. Both dominant heterozygous and recessive homozygous mutations of *AFG3L2* will lead to depletion in AFG3L2 protein. Loss of *AFG3L2* causes mitochondrial network fragmentation [49] affecting mitochondrial anterograde transport [8] but is incapable of causing any human disease [49]. *AFG3L2* is highly expressed in Purkinje cells [50] where its deficiency causes SCA28 or SPAX5 [51]. It is also expressed in the neighboring Bergmann glial cells where it plays an important role in glutamate homeostasis in the synaptic and peri-synaptic extracellular environment [52]. Ceftriaxone is a β-lactam antibiotic promoting synaptic glutamate clearance. It upregulates glutamate receptor EAAT2 in the astrocytic glial cells and ameliorates ataxia in heterozygous mutated *AFG3L2* by inhibiting glutamate excitotoxicity and creating healthy Purkinje cell and glial connections [53]. The neuron-glial cross-talk is well reported where cytodifferentiation of Bergmann glial cells proceeds in correlation to the cytodifferentiation of Purkinje cells [54]. The work of Rugarli laboratories has clearly depicted the importance of Bergmann glial cells in ataxia. Bergmann glial cells are radial astrocytes neighboring to the Purkinje cells in the cerebellum that help in clearing the glutamate toxicity at the synaptic clefts of Purkinje cells and Bergmann glial cells. This is primarily performed by EAAT1, a glutamate-aspartate transporter, and EAAT2, a sodium ion-dependent glutamate transporter. AFG3L2 deficiency in these regions has shown mitochondrial morphological changes like fragmentation but no OXPHOS dysfunction [52]. Lack of AFG3L2 also upregulates a necroptotic factor called ZBP1 leading to neuroinflammation [52, 55] and metabolic stress responses. It further affects electrophysiological balance in Purkinje cells due to increased Ca^2+^ influx causing dark cell degeneration and neuronal death of Purkinje cells [53] as a secondary effect. Thus, Purkinje cells show reduced dendrite formations and the absence of firing or excitation during depolarization. Bergmann glial cell is an active participant involved in AFG3L2 deficiency-related neurological disorders (Fig. 3).

Maltecca and co-workers have shown that *Afg3l2* missense and null mutant mice have no effect on mitochondrial protein synthesis but impact on mitochondrial energy metabolism by impaired respiratory complex I and III activity due to inadequate assembling of these complexes. This is a result of swollen and giant mitochondria with damaged cristae generated in the vacuoles of Purkinje cells as well as cell bodies of the spinal cord and dorsal root ganglia that are located near to nucleus and cell membrane [56] affecting axonal transport (Fig. 3). They have further used carbonyl formation as a marker to emphasize the importance of oxidative stress in *Afg3l2* mutants [57]. NADH dehydrogenase 1 (ND1) that causes the initiation of complex I formation is degraded in AFG3L2-dependent manner [58]. Early neuronal development in the demyelinated axons involves Nrg1-III signaling via ErbB receptors [59] while post-myelination axonic development involves phosphorylation of neurofilaments (NF) utilizing the kinase-phosphatase cycles [60]. *Afg3l2* mutants impact the axon-glial cross talk because damaged axons affect myelination, result in insufficient NF phosphorylation and also cause glutamate excitotoxicity in glial cells [56, 61].

Mitochondrial proteotoxicity is a causative agent of SCA28. Heterozygous M665R mutation of *Afg3l2* in a mice knock-in model showed enhanced Purkinje cells firing and changes in the mitochondrial energy metabolism that includes a reduction in membrane potential, oxygen consumption, and ATP synthesis leading to mitochondrial fragmentation. The fragmentation was due to the presence of excess OPA1 short forms [62]. Homozygous M665R mutation is lethal. Chloramphenicol, a mitochondrial protein synthesis inhibitor, was reported to be able to reverse these mitochondrial morphologies like fragmentation in mouse embryonic fibroblasts (MEFs) generated from homozygous M665R mutation of *Afg3l2* in mice. These morphologies include mitochondrial network formation and shapes. To date, one epigenetic case study on monozygotic twins has revealed that hypomethylation of *AFG3L2* in one of the twins maintains health while changes in methylation at multiple loci of *AFG3L2* called as differential methylation on the other caused myocardial infarction [63]. However, this observation needs further exploration.

### AFG3L2 and Cellular Respiration

In general, AFG3L2 acts as a sensor to maintain organelle fitness by regulating mitochondrial proteostasis. This is done by coordinating the OxPhos complex assemblies [12]. Under stress conditions, membrane potential is compromised leading to *OMA1* activation that cleaves OPA1 required for mitochondrial fusion. However, mitophagy due to inhibition of anterograde transport in AFG3L2-depleted cells confirms that it is a result of mitochondrial fragmentation/fission [8]. Rugarli laboratory has successfully shown the role of Afg3l2 in Purkinje cell survival by supporting mitochondrial protein synthesis. Deletion of *AFG3L2* clearly reduced only the levels of mt-encoded CYTB, COX1, COX3, and ND2 in the brains but not the levels of nuclear-encoded COX4 and NDUFA9 that are mostly related to the electron transport chain [50]. Respirasomes are supercomplexes of OxPhos present in mitochondrial inner membrane. Truncated COX1 can still interact with AFG3L2 and form unstable supercomplexes. AFG3L2 abundance or depletion can regulate the stability of the truncated COX1 and its ability to generate stable or unstable respirasomes respectively [64].

Inefficient cell respiration due to loss of *AFG3L2* cause reduced cell proliferation and defective biogenesis of cell respiratory proteins, elevated expression of OMA1, and loss of paraplegin [65]. It mainly affects the assembly and hence the function of Complex IV of electron transport chain which is the rate-limiting step of oxidative phosphorylation. This may lead to an uncoupling effect thus reducing the oxygen consumption, affecting the ATP synthesis and activating apoptosis [66] (Fig. 3). Deficiency of cytochrome c oxidase (Complex IV) causes aging and degenerative disorders. At the molecular level, reduced cytochrome c oxidase affects mitochondrial membrane potential, ATP synthesis, calcium uptake, and ROS production. Caveolin-1 acts as an important interacting protein of AFG3L2 and promotes translocation of both these proteins to the mitochondria especially after enhanced oxidative stress in fibroblasts [67]. Curbing their interaction with mutant AFG3L2 protein affects Complex IV formation after oxidative stress. Loss of caveolin-1 inhibits mitochondrial translocation of AFG3L2 thus affecting mitochondrial protein quality control, collapse of OXPHOS, and lowering of ATP production [67].

A study on primary MEFs and primary cortical neurons by Bettegazzi and coworkers in *Afg3l2*-knock out (KO) mice showed that in spite of mitochondrial dysfunction leading to increased ROS production, the cells overexpressed antioxidant peroxiredoxin 3 and had higher glutathione levels after initial vulnerability of few days. This evidently maintained the cell viability as well as the quality control of the cell and can be utilized in designing therapeutic targets against aging cells found in several neurological disorders [68].

### Mitochondrial Stress Response

m-AAA mutations in *AFG3L2* or *SPG7* lead to proteotoxic trigger due to defective mitochondrial mRNA synthesis followed by inhibited nascent polypeptide chain quality control. This leads to a stress response affecting the inner membrane morphology and homeostasis of ribosome [69]. Mitochondrial stress response is handled by i-AAAs and m-AAAs differently. AFG3L2 is the major protease involved in the i-AAA and m-AAA coordinated processing of OMA1 consequently controlling the distribution of the long (L-) and short (S-) isoforms of OPA1 [70]. i-AAAs YME1 and OMA1 separately or cooperatively control the OPA1 processing thus maintaining the long (L-) and short (S-) forms of OPA1 and this further maintains proteostasis by balancing mitochondrial fission and fusion [11, 70]. L-OPA1 is essential for mitochondrial fusion. The two forms of OPA1 is balanced based on normal and stress conditions under ATP replete or ATP depleted conditions respectively. This also affects the lamellar cristae morphology, OXPHOS function and protein synthesis (Fig. 3). Richter U and co-workers, observed for the first time, the connection between OPA1 processing and protein synthesis in mitochondria is linked to ribosomes [69]. They showed that AFG3L2 dysfunction affecting protein quality control triggers stress response by OMA1 mediated S-OPA1 formation and leads to ribosomal decay [69]. Quantitative overexpression of wild *AFG3L2* or *AFG3L2* E575Q mutation (proteolytic mutant inhibiting homo-oligomerization but maintaining AAA domain function) reduces OPA1 processing and mitochondrial ribosomal protein synthesis in *MT-ATP6* m.9205delTA mutation [69, 71]. AAA domain of AFG3L2 with chaperonic function has epistatic suppression effect on *MT-ATP6* m.9205delTA non-stop mutation during heat shock stress [69]. *AFG3L2* mutations lead to enhanced OMA1-dependent S-OPA1 formation and mitochondrial fragmentation [72–74] that ultimately causes neurodegeneration. Overexpression of *AFG3L2* in these mutants does not rectify the aberrant protein synthesis [69].

### Substrate Identification and Processing

Specialized mechanisms are involved in substrate-specific identification, recruitment and degradation/processing of substrates but these mechanisms still largely remain unclear [31]. Interestingly, given their role in diverse cellular activities, these AAA + proteases recognize both highly specific sequences like the degron sequences and residue patterns as well as accessible sequences in unstructured regions thus performing both targeted protein degradation and untargeted degradation. Degron sequences and residue patterns following scissile peptide bond (a covalent chemical bond) region are the two ways by which m-AAA proteases maintain their specificity. For example, hydrophobic and small polar residues in P1 position at N-terminal side of substrate MrpL32, a mitochondrial ribosome act as a degron that interacts with AFG3L2 for degradation [29, 75]. The loss of AFG3L2 (m-AAA) and YME1L (i-AAA) results in mitochondrial fragmentation and impairs cell respiratory biogenesis [65]. Accurate mechanisms by which AAAs precisely identify the degron sequences still largely remain unexplored for majority of their substrates.

### MCU Complex Assembly and Calcium Homeostasis

m-AAA like AFG3L2 are also involved in mitochondrial calcium uniporter (MCU) complex assembly [4]. Mutations of *AFG3L2* affect this assembly leading to Ca^2+^ overload, increased reactive oxygen species (ROS) production due to elevated cellular respiration thus releasing proapototic factors like cytochrome C into the cytosol. Purkinje cells are prevalently affected in ataxias due to the imbalance in their calcium ion reservoir which in general is linked to calcium channels, calcium interacting protein, calcium-related phosphatases, and kinases [76]. Mitochondria, due to its close proximity to both endoplasmic reticulum (ER) and cell membrane promotes calcium influx from ER and cell membrane into the mitochondria, thus act as a calcium buffering system. At neuronal synapses, this affects the neurotransmitter release especially in Purkinje cells that cause an elevated firing rate [4].

Ca^2+^ homeostasis is majorly affected in Purkinje cells that have highly branched dendrites and receive excitatory inputs through glutamate stimulated receptors AMPA and mGluR1 [77]. This excitation increases the Ca^2+^ uptake in these cells. Lack of AFG3L2 not only causes mitochondrial fragmentation, it possibly keeps mitochondria available and functional in the soma of the Purkinje cells close to ER, thus making less mitochondria and ATP available in the axons and dendrites [61]. This restricts the ATP-dependent Ca^2+^ uptake in these major excitatory regions. This may lead to glutamate toxicity leading to apoptosis of these cells causing neurodegeneration.

*LACE1*, the human homolog of yeast *Afg1* (mice *Afg3l1*), deletion causes apoptotic resistance while *LACE1* overexpression enhances apoptosis [78]. It helps in the mitochondrial translocation of p53. However, such evidence is not yet recorded in AFG3L2. *AFG3L2* deletion causes mitochondrial fragmentation while deletion of both *AFG3L1* (another m-AAA complex component) and AFG3L2 causes oligodendrocyte/glial death due to demyelination and motor dysfunction including hair graying [49]. Konig and Rugarli’s work from Langer laboratory found that loss of m-AAAs generates constitutively active MCU-EMRE channels that cause calcium overload in mitochondria affecting permeability and leading to cell death [41, 54].

MCU complex includes pore-forming MCU unit, regulatory proteins MICU1 or MICU2, and MCU interacting protein EMRE [41, 79–81]. The active MCU includes all of these components, and its assembly is brought about by m-AAAs. In the absence of the regulatory protein (MICU1/MICU2) or its interaction with EMRE, that is required prior to the complete complex assembly, MCU and EMRE assemble into a constitutively active complex that disrupts calcium homeostasis and brings in apoptosis [41]. This can be the reason for neurodegeneration and muscle disorders. *MCUb* in mice is a variant found as a dominant negative form [82]. It is reported that the absence of AFG3L2 increases the cytosolic calcium influx in the Purkinje cells causing ataxia due to constitutively active MCU-EMRE assembly [4]. It is noteworthy to mention that *MICU1* deletion results in the development of ataxia and muscle weaknesses [80]. Excitatory increase in mitochondrial Ca^2+^ causes SCA28 by Purkinje cells apoptosis [80]. EMRE acts as a novel substrate of AFG3L2, and its regulated proteolysis maintains Ca^2+^ homeostasis. AFG3L2/paraplegin duo degrades unassembled EMRE utilizing ATP breakdown [6]. The study of regulation of Ca^2+^ homeostasis by AFG3L2 can identify novel MCU inhibitors as prospective therapeutic intervention against the apoptotic process. While in one hand m-AAA regulate MCU complex assembly and hence the mitochondrial calcium uptake, on the other hand, they regulate mitochondrial protein synthesis and further influence ROS production and cellular death by apoptosis [4]. Maintenance of proteostasis is a multi-step process including many mechanisms, and hence, inhibition of mitochondrial Ca^2+^ uptake may not lead to Purkinje cell survival as per the studies undertaken by Langer laboratory [4, 41].

Maltecca and co-workers have pinpointed that loss of *Afg3l2* in MEFs causes primarily respiratory dysfunction leading to mitochondrial fragmentation and enhanced OPA1 processing that affects calcium homeostasis. The lack of calcium diffusion into mitochondria is clearly due to the impaired cross-talk between the ER and the mitochondria that can be retrieved by OPA1 or MFN1 overexpression. However, this approach also exhibit continuing respiratory defects [61]. Later, Maltecca’s laboratory and Tempia’s laboratory have proven in SCA28 cells that OMA1 hyperactivation followed by increased processing of OPA1 causes defective mitochondrial fusion due to the accumulation of mtDNA-encoded proteins [62, 83]. This also reduced the mitochondrial calcium uptake.

## Ataxia

National Ataxia Foundation of America reports that approximately 150,000 Americans are affected with sporadic or hereditary ataxia. People of any age or sex can suffer from ataxia, a slowly progressive neurological disorder. Ataxia is an uncontrolled movement disorder caused by the improper functioning of the nervous system. There are three kinds of ataxia: vestibular ataxia, spinocerebellar ataxia, and proprioceptive or sensory ataxia [84]. These are also categorized as vestibulocerebellar, cerebellar motor, and cerebellar cognitive syndromes based on their neuroimages and anatomical findings [85]. Vestibular ataxia develops due to impaired functioning of vestibular system, which senses movement of head and help maintain balance and spatial orientation. It involves the inner ear and the ear canals. Abnormality in nerves of vestibular system manifests the following symptoms: blurred vision, nausea and vomiting, problems with standing and sitting, trouble in walking, virtigo, or dizziness [86]. Spinocerebellar ataxia develops due to impaired functioning of cerebellum or spinal cord, which helps maintaining balance and coordination of body movements [87]. Symptoms of cerebellar ataxia include headache, changes in voice, slurred speech, fatigue, muscle tremors, dizziness, trouble walking, and wide gait. Proprioceptive or sensory ataxia develops due to impaired functioning of the nervous system that is outside of the brain and spinal cord, which helps maintain touch sensation of the skin. Symptoms of sensory ataxia include difficulty touching finger to nose with closed eyes, inability to sense vibrations, trouble walking in dim light, and walking with a heavy step [84].

Inherited ataxias can be autosomal dominant or recessive, X-linked, and episodic ataxia [85]. While dominant ataxia is easily diagnosed with prominent phenotypes, recessive ataxia shows a high heterogeneity in its phenotypic expression and X-linked ataxia are best identified from trios studies. Recessive ataxia is often initiated in childhood or early adulthood [85]. Strupp laboratory categorizes the phenotypic expressions of autosomal recessive cerebellar ataxias (ARCAs) into six categories but AFG3L2 related SPAX5 falls under metabolic or mitochondrial syndrome [85]. Difficulties in diagnosis and management are more evident in identification of autosomal recessive ataxia where combined results of next generation sequencing and trios studies help in accurate diagnosis and detection [88]. In the past 4 years, 15 cases of early onset cerebellar ataxia have been diagnosed in Kasturba Hospital, Manipal, India. Indian prevalence in cerebellar ataxias is reported as 4.8 to 13.8 in 100,000 individuals [89]. SCA28 is a rare type of ataxia, and *AFG3L2* has been shown to be involved with this disorder in several studies across the world [22, 29, 83, 90–92]. However, the molecular mechanisms are yet to be deciphered.

So far, some mechanisms related to *AFG3L2* mutagenesis that contributes to SCA28 pathophysiology have been reported. Being a m-AAA protease in the inner membrane of mitochondria, AFG3L2 is involved in mitochondrial proteostasis and cellular respiratory biogenesis [65]. Loss of these AAA proteases can affect protein synthesis and cellular respiration [4]. It is highly expressed in GABA-ergic Purkinje cells of cerebellar cortex [51] and involved in the axonal anterograde transport of mitochondria [8]. The neuronal interactome of AFG3L2 includes paraplegin, PINK1, EMRE, and MAIP1 as the prevalent interaction proteins [4]. Since *AFG3L2* demonstrates both autosomal dominant and recessive mutational inheritances, it adds on to the diagnostic dilemmas at both allelic and phenotypic aspects of SCA28 or SPAX5 development and gives added challenges to genetic counselling.

## *AFG3L2* and Its Related Mutations in Ataxia

AFG3L2 is a mitochondrial inner membrane zinc metalloprotease, essential for axonal transport of mitochondria and neuronal development [4], required for paraplegin and PINK1 maturation [93] as well as has chaperonic function of ATPase domain [11]. *SPG7* mutations cause autosomal recessive hereditary spastic paraplegia (HSP type 7) affecting Complex I activity and oxidative stress [94, 95]. While autosomal dominant *AFG3L2* mutations cause SCA28, homozygous recessive mutations lead to SPAX5 [12, 19, 24]. Disease-relevant *AFG3L2* mutations are localized in four hotspots of which three hotspots categorized as autosomal dominant mutations are found in three inter-subunit interfaces while all recessive mutations are found in the peripheral active sites of protease ring [36] (Fig. 1). These mutations derange the nucleotide dependent substrate translocation affecting the substrate interacting non-conserved regions. All protease domain recessive mutations except N435T condense at the central protrusion [36]. However, these spinocerebellar ataxia of autosomal recessive types abbreviated as SCAR are difficult to identify and their mechanisms remain less explored and elusive [96]. The domain structure of AFG3L2 listed in Table 1 and Fig. 1 gives us a clear depiction of how domains can be related to the neurological disorders. We can see that mutations in the catalytic domain cause optic atrophy or OPA12 while the pro-peptide, inter-TM1 (Trans-membrane region 1), IMS (Inner membrane space), and proteolytic domain mutations are mainly responsible for SCA28. SPAX5, being a recessive phenotype, is rarely expressed and hence is less documented, but mutations are found to be scattered in various domains inter-TM1, IMS, and proteolytic domains.

**Table 1:** The domains and amino acid position of *AFG3L2* mutations with related disorders (SCA28 (spinocerebellar ataxia 28), SPAX5 (spastic ataxia 5), optic atrophy, and SCAR (spinocerebellar ataxia autosomal recessive)). The mutational change in genomic sequence is documented where available. *Yet to be determined

Domain	Amino acid position	Disease	References
Pro-peptide	R41*	SCA28	[140]
In b/w pro-peptide and TM1	P78S	SCA28	[96]
	E98Q	SCA28	[96]
	G116R *c.346G* > *A*	SCA28	[13]
	W128* *c.571G* > *A*	SPAX5	[96]
IMS	V191I	SCA28	[140]
	I216F	SPAX5	[96]
In b/w catalytic and TM2	K306E *c.916A* > *G*	Optic atrophy	[140]
	G337E	Optic atrophy	[116]
	A338T	Optic atrophy	[140]
Catalytic domain	L346F *c.1036C* > *T*	Optic atrophy	[140]
	T355M *c.1064C* > *T*	Optic atrophy	[140]
	L356R	Optic atrophy	[140]
	E376K	Optic atrophy	-
	F377S *c.1130 T* > *C*	Optic atrophy	[140]
	D407G *c.1220A* > *G*	Optic atrophy	[140]
	D411A	Optic atrophy	[13]
	R416A	SCA28	[27]
	T430I *c.1289C* > *T*	SCA28	[140]
	N432T	Optic atrophy	[140]
	A462V *c.1385C* > *T*	Optic atrophy	[140]
	R465K *c.1394G* > *A*	Optic atrophy	[140]
	P466L *c.1402C* > *T*	Optic atrophy	[140]
	R468C	Optic atrophy	[140]
	P477L	SCA28	[96]
	A484P	SCA28	[96]
	P514L *c.1541C* > *T*	Optic atrophy	[140]
Proteolytic domain	L556fs *c.1714G* > *A*	SCA28	[96]
	A572T	SPAX5	[140]
	E575Q	SCA28	[108]
	Y605C *c.1847A* > *G*	Optic atrophy	[140]
	Y616C	SPAX5	[140]
	Q620K *c.1858C* > *A*	SCA28	[140]
	L621V *c.1875G* > *A*	SCA28	[140]
	M625I *c.1894C* > *T*	SPAX5	[96]
	R632*	SCAR	[96]
	S634* *c.1901_1902delCT*	Optic atrophy	[140]
	T654fs *c.1961C* > *T*	SCA28	[96]
	T654I	SCA28	[140]
	F664S	SCA28	[140]
	G665V *c.1997 T* > *C*	SCA28	[140]
	M666T/V/R *c.1996A* > *G M666V; c.1997 T* > *G M666R*	SCA28	[140]
	G671W/R/E *c.2011G* > *A (G671R)*	External ophthalmoplegia/SCA28	[140]
	S674L	SCA28	[140]
	K687E *c.2062C* > *A*	SCA28	[140]
	P688T	SCA28	[140]
	P688A	SCA28	[140]
	Y689H/N	SCA28	[140]
	E691K	SCA28	[140]
	A694E	SCA28	[101]
	R695G	SCA28	[140]
	E700K	SCA28	[140]
	R702Q	SCA28	[140]
	I705T *c.2167G* > *A*	SPAX5	[140]
	V723M	SCAR	[96]
After proteolytic domain	E793* *c.2375dupG*	Optic atrophy	[96]

## Mutations in Spinocerebellar Ataxias

Spinocerebellar ataxias (SCA) or autosomal dominant cerebellar ataxia (ADCA) are rare autosomal dominant progressive neurological disorders characterized by gait imbalance and motor incoordination of hand, speech, and eye movements—a primary cerebellar dysfunction. By 2010, although over 30 SCA genes were identified that are related to these disorders, the cellular and molecular events have not been completely deciphered yet [97]. 18p whole arm translocation was reported to be causing dystonia with symptoms of rigid postures and muscle contractions [98]. In 1999, *AFG3L2* was mapped at location 18p11, and further, the locus of SCA was identified in 2006 where specifically 18p11.22-q11.2 was designated as SCA28 [24–26]. Haploinsufficiency has also been reported to cause SCA28 [99]. A detailed list of mutations in AFG3L2 and their relation to domain structures can be seen in Table 1 and Fig. 1. The first case of heterozygous deletion of *AFG3L2*, a causative reason for SCA28 due to haploinsufficiency which has multiple genomic anomalies, was reported in 2014 [100]. However, Lohmann laboratory studying SPAX5 related mutations had previously stated that heterozygous *AFG3L2* mutation causes SCA28 [101]. *AFG3L2* mutations, although are related to cerebellar ataxias, does not exhibit polyglutamine repeats like the majority of SCA genes [99]. Patients with no signs of prevalent SCA types are screened for SCA28 and symptoms include oculomotor signs of a very slowly progressive ataxia [25]. Cagnoli and co-workers [102] reported six missense mutations of *AFG3L2* in nine unrelated index cases from 366 European families having ADCA (autosomal dominant cerebella ataxia). Similarly, in a Taiwanese cohort, only one patient was reported for *AFG3L2* mutation among 133 cerebellar ataxia patients. In another ADCA cohort study, Jia et al. [91] did not find any *AFG3L2* mutations from 67 patients. In 2013, a study on the lymphoblastoid cell lines of four SCA28 patients revealed 66 genes with statistically different expression patterns [103]. This was the first genome-wide analysis that identified 35 upregulated and 31 down-regulated genes that were categorized into five functional categories that are related to cell proliferation, programmed cell death, oxidative stress response, cell adhesion, and chemical homeostasis. The differential expression is related to phenotypes of SCA28 that include impaired growth, increased G0/G1 phase cells, increased apoptosis, enhanced lipid peroxidation, and increase in mitochondrial regulators TFAM and DRP1 but no alteration in ROS levels and respiratory chain activity.

*AFG3L2* gene with 17 exons encodes a 797 amino acid protein. By 2010, *AFG3L2* gene mutations was identified to cause SCA type 28 [90]. It has been reported that exons 15 and 16 are the mutational hotspots for *AFG3L2* for causing SCA28 [23, 102]. AFG3L2 also has selective overexpression in the Purkinje cells and many *AFG3L2* dominant mutations as discussed by Di Bella D and coworkers’ results in SCA28 [51, 87]. Y689H substitution in the M41 peptidase domain was reported as pathogenic and to cause SCA28 [87]. First missense mutation in exon 4 (not the mutation hotspot) of an African origin patient, V191I [c.571G > A], was identified in a 68-year-old patient [104]. But, it could not be identified whether this was a founder mutation. A novel heterozygous partial *AFG3L2* deletion of exons 14 to 16 was reported to cause loss of cerebellar function with ptosis [105]. The disease mechanism was related to ubiquitin and p62 nuclear inclusions and haploinsufficiency. Thirteen missense mutations of *AFG3L2* mostly in exon 16 have been identified till date, but cases are rare in Western countries [92]. However, all mutations discovered and documented so far are mostly in Caucasian populations. A novel G671R [c.2011G > C] mutation that was located in a highly conserved region of *AFG3L2* gene in five patients of a Hungarian family showed similar characteristics to previously identified cases with no cognitive impairment [106] and included pathogenic mutations G671R [c.2011G > A] and G671W [c.2011G > T] [102, 107]. A rare compound heterozygous mutations of *AFG3L2*, Y616C [c.1847A > G], and V723M [c.2167G > A] caused ptosis in his son as well as his asymptomatic single heterozygous mutated mother [103]. This mutation can act as a pre-diagnostic marker for future case detections. Such compound heterozygous mutation adds more to mitochondrial dysfunction both structurally and phenotypically. Heterozygous loss-of-function mutations in *AFG3L2* cause SCA28 while homozygous missense mutation Y616C [c.1847A > G] in *AFG3L2* causes early onset spastic ataxia-neuropathy syndrome also known as SPAX5 [23]. p.A484P variant of *AFG3L2* segregated with STIP1 homology and U-Box containing protein 1 (STUB1) variant (Y49C) that causes SCA16 and SCA48 [108]. A Taiwanese patient showing multiple heterozygous mutations, i.e., [2167G > A]; [V723M] (c.[1894C > T]; [R632*], suffered from sporadic and slow progressive SCA [96] and represented a new subtype. Maltecca and co-workers [99] have identified two mutations—homozygous missense and homozygous null—that caused lethality in mice models due to impaired axon development in both CNS and PNS delayed myelination, and weak axonal radial growth [56]. In *Spg7* deficient mice, Emv66 mutants of *AFG3L2* show exacerbating axonopathy and severe neuromuscular defects in hetero- and homozygous conditions respectively with loss of PCs and parallel fibers [109]. Heterozygous P688T [c.2062C > A] mutation at highly conserved site in exon 16 in three patients of a family for the first time reported non-neuronal skeletal muscle fiber atrophy type I along with SCA28 phenotypes [110].

## Mutations in Other Ataxias

Interestingly, SCAR mutations cause spastic ataxia-neuropathy syndrome or SPAX5 [23]. Purkinje cell-specific deletion of *Afg3l2* leads to mitochondrial fragmentation due to impaired mitochondrial ribosome assembly and protein synthesis [50]. However, ubiquitous *AFG3L2* deletions lead to delayed myelination causing lack of axonal development and a severe neurological phenotype [56].

Tyrosine phosphorylation of paraplegin by AFG3L2 helps in paraplegin processing and inhibition of this processing can cause ROS production [111]. On the contrary, the Q688 variant of paraplegin can bypass the tyrosine phosphorylation regulation of AFG3L2 [112]. Another type of SCAR found by Calandra and co-workers includes two novel *AFG3L2* heterozygous mutations (W128* and R695G) that cause spastic ataxia with unique eye-of-the-tiger pattern that can be related to iron deposition and pantothenate kinase-related neurodegeneration that affects CoA synthesis pathway [18]. Long-term observation will be required to see the development of SCA28 phenotype if any. Yeast homolog *Yta10* of *AFG3L2* is reported to mislocalize during hypoxia [113] and triggers the question about the role of AFG3L2 in the proteostasis of mitochondrial proteins and the onset of SCA28.

Heterozygous Y689H and G671W mutations of *AFG3L2* cause progressive external ophthalmoplegia due to somatic mtDNA disbalance affecting fibroblasts and muscles of the eye [107]. *AFG3L2* was identified as one of the brain-expressed longevity genes [114], and further, SNPs in *AFG3L2* were identified relating to aging [115]. Heterozygous R468C mutation along with heterozygous *SPG7* deletion leads to aberrant OPA1 processing. Since OPA1 is involved in mitochondrial fusion, this mutation further causes mitochondrial fragmentation. Phenotypic characterization is expressed as early-onset optic atrophy associated with Parkinsonism (L-Dopa responsive) and spastic ataxia [22]. G337E [c.1010G > A] mutation of *AFG3L2* causes aberrant processing of OPA1 and OMA1 leading to optic atrophy [116]. Homodimer of AFG3L2 causes better OPA1 processing and continues this process even in the absence of heterodimer with paraplegin or presenilins-associated rhomboid-like protein (PARL) [117]. Involvement of OPA1 processing with various dimer formations of m-AAAs provides flexibility in mitochondrial biogenesis in a tissue/ function-specific manner and therefore improves the quality of life [30]. Thus, AFG3L2 clearly plays a vital role in the prevention of neurodegeneration in the cerebellum by regulating mitochondrial proteostasis and restricting apoptosis. More investigation is required to understand this mechanism.

## Diagnosis, Management, and Treatment of SCA28

Diagnosis and management of cerebellar ataxia needs a multidisciplinary approach with neuroimaging, anatomical studies, genetic tests, motor tests, and molecular/biochemical studies playing crucial roles [85]. Since SCA28 is a slowly progressive, young-adult onset ataxia that in most of the cases may take decades to be identified, the diagnosis usually is restricted to a proband whose either parent was affected and hence can be diagnosed by genetic testing via exome sequencing for the typical pathogenic variant as discussed in this review article. These tests necessarily need to include multigene panel and comprehensive genetic analysis. Till date, exome or genome sequencing is not yet available routinely for SCA28 patients [118]. Clinical exome sequencing is the most efficient and economical way to diagnose the rare pathogenic variants and to discover likely variants of *AFG3L2* [119]. The presence of the *AFG3L2* mutations through family history also becomes challenging due to delayed diagnosis or the fact that probably it remained undetected due to the early demise of the parent [118, 120]. Neuroimaging may sometimes show a shrunken cerebellum [118] but mostly show a normal neuroimaging in MRI/CT scan [121]. Hence, a keen investigation with family history, physical examinations, genetic testing, and neuroimaging might be useful in diagnosing this disease. Very recently, c.2167G > A; V723M variant is designated as a likely pathogenic variant of *AFG3L2* that is related to myopathy, respiratory chain complex defects, and ataxia [122] in a single patient who is also affected by congenital pituitary hormone deficiency and deafness due to involvement of other mutated genes as well.

There is a complete lack of treatment for SCA28 primarily because of its slow progression and difficulty in timely diagnosis. Hence, it is of utmost importance to manage this disorder well with timely interventions whenever diagnosed. These interventions include ambulatory aids, stretching exercises, and physical therapy; ergonomic home adaptations; speech and psychological therapies; weight control assessment; and gastronomic feeding—all or as many of these as required along with annual assessment [118]. Very recent studies have identified an important variant of the nuclear-encoded *AFG3L2* with a SNP affecting mRNA half-life and associated with exercise response phenotypes [123].

## Other Clinical Manifestations of *AFG3L2*

### Spastic Ataxia Syndrome (SPAX5)

SPAX5 or spastic ataxia type 5 is a SCAR caused by the homozygous recessive missense mutations of *AFG3L2*. This syndrome is an early-onset type with cerebellar dysfunction but is characterized by spasticity and epilepsy. Since *AFG3L2* mutations affect both homo- and hetero-hexamerization of AFG3L2, it includes the severity of both SCA28 and spastic paraplegic ataxia due to the involvement of SPG7 in hetero-hexamerization. AFG3L2 dysfunction can severely damage mitochondrial functioning causing massive fragmentation and reduced calcium uptake [83]. The mutation specifically at Y616C [c.1847A > G] of *AFG3L2* leads to phenotypically different manifestations from SCA28 and causes spastic ataxia [23]. However, homozygous mutations such as W128, I216F, and I705T also cause SPAX5 and are listed in Table 1, and compound heterozygous mutation can cause SPAX5 too. Compound heterozygous Y616C [c.1847A > G] with p.V723M [c.2167G > A] mutation [23] also is reported to cause SCAR. SPAX5 manifestation shows a severe reduction in AFG3L2 expression in a case study with biallelic compound heterozygous p.V212Gfs*4 [c.634dupG] with p.V723M [c.2167G > A] mutation [124]. The severity of this syndrome may surely affect other genes since it is the outcome of dysfunction of m-AAA proteases. Homozygous M625I [c.1875G > A] mutation is reported as a rare mutation of *AFG3L2* related to progressive myoclonus epilepsies [125, 126].

### Parkinson’s Disease

Unique frameshift *AFG3L2* mutation, c.1958dupT in exon 15, causes mild Parkinsonism with cognitive decline, cerebellar ataxia, bradykinesia, and polyneuropathy [127]. The direct relation of this mutation with the phenotype of the patient is currently unclear, and this being a case study shows a weak association with PD. However, AFG3L2 haploinsufficiency and surely not the dominant-negative effect of missense AFG3L2 mutation reported in SCA28 is likely the reason for this pathogenesis [127]. AFG3L2 aids in PINK1 maturation by generating a 52kD processed fragment that can localize in IMM and hence may indirectly be linked to PD. PINK1, a protein associated with the elevation of autophagy, is synthesized in cytosol and interacts with alpha-synuclein [128]. Cofilin, an actin-binding protein, mediates PINK1/PARK2-dependent mitophagy [126]. PINK1 accumulation due to dysregulation of AFG3L2 and mitochondrial membrane potential [129] can affect this process leading to cancer progression. PINK1 undergoes a stepwise degradation from cytosol to mitochondrial matrix that is controlled by MPP, PARL, AFG3L2, and ClpXP respectively [93]. Under stress conditions, PINK1 which is partially cleaved by mitochondrial processing peptidase (MPP) is transported to IMM and undergoes presenilin-associated rhomboid-like protein (PARL) and AFG3L2-dependent processing forming a 52 kD fragment [15, 130]. During membrane depolarization or ATP depletion, the absence of PINK1 cleavage by MPP recruits uncleaved PINK1 to the outer mitochondrial membrane (OMM). PINK1 further recruits Parkin, a E2 ubiquitin ligase causing the mitochondrial fragmentation of damaged mitochondria [3]. A well-known fact today is that the loss-of-function of Parkin leads to PD, both due to the absence of PINK1-Parkin interaction and alpha-synuclein accumulation [131, 132]. Whether *AFG3L2* mutations causing SCA28, SPAX5, or optic atrophy can cause additional risk of PD by generating altered PINK1 cleavage is an intriguing aspect to test. Mendelian disease mapping in a family by pedigree analysis must be performed to establish causation. AFG3L2 loss leads to tau hyperphosphorylation [8] although the mechanism behind its relation to AD is not yet studied. Mutation in *PARK8* gene locus encoding LRRK2 plays a vital role in PD [133].

### Mitochondrial Disease

In a case study including a consanguineous family, c.1714G > A A572T mutation on *AFG3L2* was identified in the first-ever exome sequencing analysis to be involved in a new mitochondrial phenotype that caused high lactate and respiratory chain defects [134]. Cerebellar atrophy was noticed in the neuroimaging. In a detailed study, this mutation was seen to cause microcephaly with early onset seizures and involves basal ganglia that are not noticed in other mutation types. This has further led to refractory epilepsy and death. This study emphasizes that this *AFG3L2* mutation can be added as a genetic marker along with neuroradiological and biochemical spectrum for a rare mitochondrial disease with neurodegenerative phenotype.

### *Optic Neuropathy* and Other Eye Associated Ataxias

Optic neuropathy or dominant optic atrophy (DOA) is a dominant and inherited mitochondrial disease majorly caused due to mutations in OPA1. The cleavage of this protein is dependent on i-AAA YME1L and m-AAAs AFG3L2 and paraplegin. *SPG7* mutations are more often related to this disease. Other mutations include OPA3 [135, 136], OPA5 [137], and WFS1 [138]. c.1402C > T R468C in *AFG3L2* was identified as a novel mutation in two patients in a family from two generations who additionally had dyschromatopsia but no sign of either SCA28 or SPAX5 [9, 13, 14]. It is possible that these patients may develop cerebellar ataxia later in their life. In that case, this mutation can act as a pre-diagnostic marker for SCA28 or SPAX5 and surely as a marker for optic neuropathy-related blindness. It will also be interesting to find whether this mutation is present in any SCA28 or SPAX5-affected patients. Eight variants of *AFG3L2* responsible for DOA are located in different domains and establish *AFG3L2* as a candidate gene for DOA detection [139].

Caporali and co-workers performed an exhaustive study on the role of AAAs in optic neuropathy where their studies showed using both yeast and patient fibroblast that *AFG3L2* mutations indirectly affect OPA1 processing, resulting in mitochondrial fragmentation not seen in SCA28 patients. This has been proposed as the crucial mechanism behind the pathogenicity for the studied variants. It clearly summarized 38 *AFG3L2* variants in patient cohorts from their own as well as other studies where pathogenic variants related to optic neuropathy were clustered in the ATPase domain affecting either of the functions related with ATP hydrolysis, ATP binding, and substrate interaction. The rest of the variants associated with SCA28 and SPAX5 were clustered in the proteolytic domain [140]. The patient cohort that was studied included 12 different families with familial or sporadic mutations and followed for two to five generations to understand the genetic mechanism. Mutations of *AFG3L2* related with DOA are varied and include dominant, recessive, and dominant de novo mutations involved in both sporadic and familial cases and can be pure or syndromic forms. Optic atrophy-12 (OPA12) is an autosomal dominant neurologic disorder caused by *AFG3L2* mutation in 18p11.21 location [140]. A heterozygous *AFG3L2* mutation together with a homozygous *SETX* mutation causes AOA2 (ataxia with oculomotor apraxia type 2) with myoclonus [141]. This further corroborates the potential of *AFG3L2* as a prognostic and/or diagnostic marker for various mitochondrial diseases and neurodegenerative disorders related to aging.

## Conclusion

AFG3L2, a AAA protease, is an ATP-dependent proteolytic complex which is involved in the mitochondrial quality control and protein processing. Two isoenzymes are found in human AAA protease—(i) a complex made up of paraplegin and AFG3L2 which is a hetero-oligomeric complex and (ii) a homo oligomeric complex consisting of AFG3L2. Hereditary spastic paraparesis (HSP) is mainly associated with the dysfunction of paraplegin, whereas mutation of *AFG3L2* is involved with primarily causing SCA28. Most of the studies imply that *AFG3L2* mutations are majorly linked with autosomal dominant SCA28, and some mutations are involved in developing the SPAX5 or SCAR and optic atrophy. As several mutations are reported in exons 15 and 16 repeatedly, these exonic regions are considered the hotspots of SCA28 pathogenesis. Trios studies for genetic analysis are highly recommended as routine diagnosis during pregnancy and in infant check-up sessions to enable the early detection of the at-risk asymptomatic individuals. Detection of recessive mutations in *AFG3L2* would be possible only by genetic testing for cases like a child born to asymptomatic parents, adopted child, paternity issues, and maternity issues in case of surrogate motherhood. As AFG3L2 is mainly associated with the mitochondrial biogenesis and dynamics, its malfunction leads to a variety of neurodegenerative disorders. The effect of the absence of AAA proteases on the metabolic or mitochondrial mechanisms in neuronal dysfunctions is poorly studied. This study explores the future prospects of AFG3L2, as well as its roles in the development of diseases and their therapies. Finding out the molecular basis of the pathophysiology of neuronal disorders should be a major goal to understand the underlying mechanisms. Hence, more studies are required on *AFG3L2* and its mutations.

## Data Availability

Not applicable.

## Abbreviations

AAA: ATPase associated with diverse cellular activities
ADCA: Autosomal dominant cerebellar ataxia
AFG3L2: ATPase family gene 3-like 2
AMPA: Amino-methyl phosphonic acid
AOA2: Ataxia with oculo-apraxia type 2
ARCA: Autosomal recessive cerebellar ataxia
ANS: Autonomic nervous system
ATP: Adenosine triphosphate
CHOP: C/EBP homologous protein
CNS: Central nervous system
COX 1/ 2: Cytochrome C oxidase subunit 1/2
CT Scan: Computed tomography scan
CYTB: Cytochrome b
DOA: (Autosomal) Dominant optic atrophy
EM: Elecron microscope
EMRE: Essential MCU regulator
ER: Endoplasmic reticulum
ErbB: Erythroblastic oncogene B
GABA: Gamma-aminobutyric acid
IMM: Inner mitochondrial membrane
IMS: Inner membrane space
LACE1: Lactation elevated protein 1
LON: Protease La
LRRK2: Leucine-rich repeat kinase 2
m-AAA: Mitochondrial AAA
MCU: Mitochondrial calcium uniporter
MRI: Magnetic resonance imaging
MAIP1: M-AAA protease interacting protein 1
MAPK: Mitogen-activated protein kinase
MEF: Mouse embryonic fibroblast
mito-DLP1: Mitochondrial dynamin-like protein 1
MPP: Mitochondrial processing peptidase
mt: Mitochondrial
mTOR: Mammalian target of rapamycin
mtUPR: Mitochondrial unfolded protein response
ND 1/2: NADH dehydrogenase 1/2
NDUFA9: NADH ubiquinone oxidoreductase subunit A9
NF: Neurofilament
NMR: Nuclear magnetic resonance
OMA1: Overlapping with m-AAA protease 1
OPA: Optic atrophy
OXA1L: Oxidase assembly protein 1 long form
OXPHOS: Oxidative phosphorylation
PARL: Presenilins-associated rhomboid-like protein
PD: Parkinson’s disease
PINK1: PTEN-induced kinase 1
PNS: Peripheral nervous system
ROS: Reactive oxygen species
SCAR: Spinocerebellar ataxia, autosomal recessive
SCA28: Spinocerebellar ataxia type 28
SETX: Senataxin
SOR: StAR overload response
SPAX5: Spastic ataxia 5
SPG7: Spastic paraplegia type 7
SRH: Second region of homology
StAR: Steroidogenic acute regulatory protein
STUB1: STIP1 homology and U-box containing protein 1
TM1/2: Trans-membrane region 1/2
WFS: Wolframin ER transmembrane glycoprotein
YME1L: YME1 like 1 ATPase
Nrg1-III: Neuregulin 1 type III
HCLR: HslU/ClpX, ClpABC-CTD, Lon, and R
